# Some Scientific Aspects of Insanity

**Published:** 1892-03-19

**Authors:** 


### SOME SCIENTIFIC ASPECTS OF INSANITY.

### XVII.—THE INSANITIES OF THE PUERPERAL STATE.

THE process of reproduction is the most exhaustive and trying ordeal through which the various forms of life on this planet have to pass, and in the female of the higher animals who have to carry, bring forth, and nourish their young, the ordeal is particularly exhausting. The human female in common with the higher animals is equally exposed to this physical reduction and exhaustion, but in addition, on account of the much more extensive and delicate development of the nervous system, she is more liable to serious mental derangement. The insanity of pregnancy is more interesting to, and the study of it is more adapted for the specialist and the medical practitioner than for the ordinary reader, and I shall therefore pass on to consider the more common and the more important form of insanity which supervenes shortly after parturition. The great majority of cases, and the most acute and characteristic cases, are those which occur within the first fortnight of childbirth. It is still an open question as to how much this insanity could be prevented by means of trained and skilful nursing. Puerperal insanity will occur in spite of all preventive precautions, but in a great many cases a careful observance of those hygienic instructions, the importance of which can only be fully understood and intelligently carried out by the trained nurse, will avert the fearful malady. In the great majority of cases the insanity takes the form of an acute mania, preceded sometimes by a melancholia characterised by suicidal and homicidal tendencies. The temperature ranges from 100 to 103, sometimes to 104, and the vaginal secretion becomes scanty and offensive. The patient then requires all that medical aid and skilled nursing can do for her, and in addition she requires that special nursing which her condition demands. So far as the mental nursing is concerned, the treatment is that of acute mania, or acute delirious mania. The strength requires to be maintained by the administration of suitable nutriment, and conserved by constant attention to the motor restlessness of the patient. A few of the cases have melancholia throughout, and a considerable percentage develop stupor, consequent either to the acute mania, or to the melancholia. With the aid and influence of care and suitable surroundings, it is satisfactory to know that most of the cases recover.

After prolonged nursing a mild form of melancholia, called lactational insanity, sometimes occurs in certain cases. Its treatment is simple, and is often effected by removing the patient from her surroundings, feeding her suitably and liberally, and observing generally the conditions most congenial towards convalescence from simple melancholia.

